# Molecular machinery turns full circle

**DOI:** 10.7554/eLife.70298

**Published:** 2021-06-17

**Authors:** Josep Rizo, Klaudia Jaczynska, Karolina P Stepien

**Affiliations:** Departments of Biophysics, Biochemistry and Pharmacology, University of Texas Southwestern Medical CenterDallasUnited States

**Keywords:** membrane fusion, yeast vacuoles, SNAREs, HOPS, Sec17, Sec18, *S. cerevisiae*

## Abstract

Two proteins called Sec17 and Sec18 may have a larger role in membrane fusion than is commonly assumed in textbook models.

**Related research article** Song H, Torng TL, Orr AS, Brunger AT, Wickner WT. 2021. Sec17/Sec18 can support membrane fusion without help from completion of SNARE zippering. *eLife* **9**:e67578. doi: 10.7554/eLife.67578

Many biological processes require two membranes to fuse together, such as the release of neurotransmitters from synaptic vesicles in neurons and the fusion of vacuoles in yeast. Scientists have been trying to fully understand what happens during membrane fusion for over three decades and this has led to many twists and turns, like in a mystery movie.

A protein called NSF (short for N-ethylmaleimide sensitive factor) was one of the first to be associated with membrane fusion, and it was postulated that NSF (which is the mammalian homologue of the yeast protein Sec18) was part of a 'fusion machine' made of multiple subunits (Malhotra et al., 1988). It was later found that NSF binds to sites in the membrane with the help of proteins named SNAPs (short for soluble NSF attachment proteins), which are the mammalian homologues of the yeast protein Sec17 (Whiteheart et al., 1992).

Further research led to the discovery that three proteins that had previously been implicated in neurotransmitter release were in fact SNAP receptors (SNAREs) and formed a large membrane-anchored assembly with NSF and SNAP called the 20S complex (Söllner et al., 1993b). It turned out that the three SNAREs could actually form a highly stable complex by themselves, and that this complex is disassembled after binding to SNAPs and NSF through the ATPase activity of NSF (Söllner et al., 1993a).

Two of the neuronal SNAREs that form the complex are anchored to the plasma membrane and the other to the synaptic vesicle. The SNAREs contain one or two strands of amino acids that are called SNARE motifs, which adopt helical structures as they join together to form a complex (Sutton et al., 1998; Figure 1A). The realization that the SNARE motifs bind to one another in a parallel fashion led to the idea that ‘zippering’ of the SNARE motifs, from the N-terminus to the C-terminus, draws the two membranes close together, forcing them to fuse (Hanson et al., 1997; Figure 1A).

This model was bolstered by experiments suggesting that the neuronal SNAREs can fuse model membranes called liposomes (Weber et al., 1998). Moreover, fusion in most intracellular membrane compartments was found to require similar SNARE complexes. These observations led to the textbook notion that SNARE complexes constitute the universal machine that causes membrane fusion, while NSF/Sec18 and SNAPs/Sec17 are responsible for disassembling the complex so the SNAREs can be re-used. Now, in eLife, Bill Wickner and co-workers from Dartmouth College and Stanford University – including Hongki Song and Thomas Torng as joint first authors – report new findings which suggest that this model needs to be revised (Song et al., 2021).

**Figure 1.**
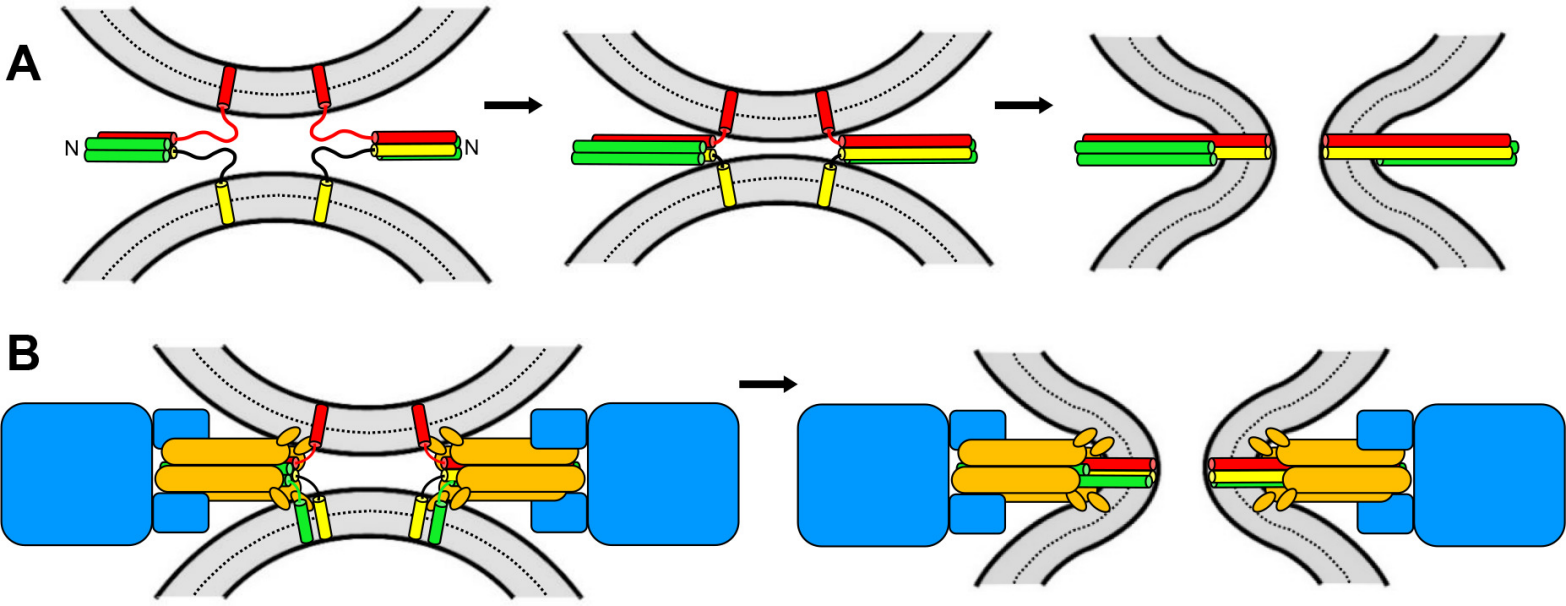
Models of membrane fusion. (**A**) Textbook model illustrated with the three neuronal SNARES: two of these (red and yellow) have one SNARE motif each and are attached to opposing membranes (shown in grey) via a transmembrane region; the third SNARE contains two SNARE motifs (green). These motifs bind together like a zipper which runs from the N-terminus to the C-terminus; this zippering process results in the formation of a four-helix bundle that pulls the two membranes together, forcing them to fuse (right). (**B**) In the model proposed by Song et al., membrane fusion relies on Sec17 (orange), Sec18 (blue) and four SNARES (one in yellow; one in red; two in green) that also form a four-helix bundle. Fusion is induced by the action of the SNAREs, as in **A**, and by the Sec17 N-terminal hydrophobic loops (small orange ellipses) perturbing the lipid bilayer of the membrane. Some of the SNAREs have N-terminal domains that are not shown for simplicity. The arrangement of the proteins is based on the structure of the neuronal 20S complex (Zhao et al., 2015).

Image credit: Josep Rizo.

Previous studies from the Wickner lab using liposomes that mimic the membranes of yeast vacuoles showed that a protein complex called HOPS helps tether liposomes together and assemble the SNARE complex (Baker et al., 2015; Mima et al., 2008). These studies also showed that the yeast proteins Sec17 and Sec18 synergize with HOPS and the SNAREs to induce fusion of liposomes. Moreover, in a remarkable twist in the plot, another group showed that Sec17 could rescue liposome fusion that was arrested because 22 amino acid residues were removed from the C-terminus of a SNARE motif (Schwartz and Merz, 2009). The removal of the residues destabilizes the SNARE complex, and hence hinders its ability to pull the two membranes together. The fact that Sec17 rescued liposome fusion under these circumstances suggests that it may have an important role in membrane fusion, beyond its involvement in the disassembly of SNARE complexes. However, it is possible that Sec17 merely helped the compromised SNARE complex to zipper fully.

Investigating this further, Song et al. revealed that Sec17 rescues liposome fusion when the C-termini of two SNARE motifs are truncated so that the zippering process is doubly hindered. Further work showed that this recovery depends on a hydrophobic N-terminal loop that Sec17 inserts into membranes, and that fusion is strongly enhanced by Sec18. Amazingly, Sec17 and Sec18 can restore fusion even when the C-termini of three SNARE motifs are impaired, showing that the SNARE complex does not need to be fully zippered in order for membranes to fuse. These findings indicate that the hydrophobic loop of Sec17 can induce fusion as long as the two membranes are brought into close enough proximity by the SNAREs.

Song et al. also found that Sec18 relies on ATP to enhance liposome fusion but without hydrolyzing ATP. This suggests that when Sec18 is bound to ATP, it joins together with Sec17 and the SNAREs to form an assembly that resembles the 20S complex, and that this most likely constitutes the true ‘fusion machine’ (Figure 1B). It is tempting to speculate that nature created this machine so that ATPase activity is slow before the membranes start to fuse; however, once fusion is complete, ATP hydrolysis speeds up to disassemble the 20S complex and recycle the SNAREs.

It now seems likely that 20S complexes mediate most types of intracellular fusion, but there may be exceptions. Indeed, a recent study showed that a mammalian SNAP stopped neuronal SNARE complexes from completing membrane fusion, suggesting that this machinery may control the fusion of synaptic vesicles in other ways (Stepien et al., 2019). It appears that the future may hold more twists and turns for this story.
